# Sex Hormones and Healthy Psychological Aging in Women

**DOI:** 10.3389/fnagi.2017.00439

**Published:** 2018-01-09

**Authors:** Esperanza Navarro-Pardo, Carol A. Holland, Antonio Cano

**Affiliations:** ^1^Department of Developmental and Educational Psychology, Universitat de Valencia, Valencia, Spain; ^2^Division of Health Research, Centre for Ageing Research, Lancaster University, Lancaster, United Kingdom; ^3^Department of Pediatrics, Obstetrics and Gynecology, Universitat de Valencia, Valencia, Spain

**Keywords:** psychological aging, women, sex hormones, cognition, mood

## Abstract

Besides their key role in reproduction, estrogens have effects in several organs in the body, as confirmed by the identification of estrogen receptors (ER) in multiple tissues. Experimental evidence has shown that estrogens have significant impacts on the central nervous system (CNS), and a key question is to what extent the fall in estrogen levels in the blood that occurs with increasing age, particularly around and following the menopause, has an impact on the cognitive function and psychological health of women, specifically regarding mood. This review will consider direct effects of menopausal changes in estrogens on the brain, including cognitive function and mood. Secondary pathways whereby health factors affected by changes in estrogens may interact with CNS functions, such as cardiovascular factors, will be reviewed as well insofar as they also have an impact on cognitive function. Finally, because decline in estrogens may induce changes in the CNS, there is interest in clarifying whether hormone therapy may offer a beneficial balance and the impact of hormone therapy on cognition will also be considered.

## Introduction

This paper sets out to review evidence on the relationships between changes associated with reductions in sex hormones in women, related to the menopause and psychological impacts, focusing on cognitive and mood changes. Beginning with cognitive effects, we examine the evidence for direct effects of circulating estrogens on neural systems, in combination with the effects of other hormones and factors that may be changing at the same time or affected by the changes in estrogens, such as gonadotropin-releasing hormone (GnRH) and insulin-like growth factor (IGF). We then examine clinical evidence from a variety of methodologies to investigate the relationships of menopausal stages, circulating hormones and hormonal therapy to cognitive changes, and dementia risk. This is followed by a similar discussion of the evidence of hormonal impacts on depression and anxiety. We finish with an examination of cardiovascular effects focusing on the impacts of cardiovascular disease on cognition, exploring the possibility that impacts of sex hormones on cognition may, in some cases, be mediated by both effects on mood and on cardiovascular health.

## Menopause and cognitive functions

Neuroscience studies have provided evidence that estrogens influence aspects of brain biochemistry and morphology known to be important for cognitive functions (Sherwin, 1997). However, while this evidence is reasonably well-supported in experimental models, relationships to neural functions in the human have been more difficult to determine because of inconsistent evidence on effects of reduced estrogens on measures of attention, concentration, or memory in clinical studies (Schmidt et al., 2013). Thus, one issue of interest is the influence of depletion of estrogens on cognition across and after menopause and, if confirmed, the mechanism through which it occurs. The potential association of menopausal hormonal changes with brain function may therefore be approached in two ways: directly, i.e., effects on neural cells and systems, and indirectly, i.e., effects of hormonal changes on evidenced functions, mainly cognition and mood (Greendale et al., 2011).

In addition, sex hormones have an influence on other systems that have impact on brain functions, specifically the vascular tree, which determines the adequacy of cerebral blood perfusion (Abe et al., 2006; Izumi et al., 2006). Observed evidence of the impact of changes in estrogens on the vascular system are obvious in common menopausal symptoms such as hot flushes, and research in this area suggests that vasomotor symptoms might represent a female-specific risk factor for memory declines during the menopausal transition (Maki, 2015).

## Direct influence of circulating estrogens on neural systems

The background supporting the action of estrogens arises from molecular evidence and observations obtained in different experimental models. There is, for example, an abundance of estrogen receptors (ERs) at several locations related to cognition in the CNS. This is particularly observed in areas related to verbal memory and retrieval, working memory, executive function, and control of attention, such as the hippocampus and the prefrontal cortex (Sherwin, 2006; Pompili et al., 2012).

There is a considerable body of knowledge explaining how estradiol exerts neuroprotective actions (Arevalo et al., 2015). The action of estrogens may occur via the increase in the levels of neurotransmitters, which may enhance neuronal growth and formation of synapses (McEwen, 2001; Hesson, 2012). In this context, cell culture experiments have shown that estrogens alter synaptic circuitry in the hypothalamus, hippocampus, and, more recently, neocortex (Hara et al., 2015). Of further importance, recent work has shown that the local production of estradiol by neural cells, as a result of the activation of the widely available aromatase in the brain, may further help in maintaining neuroplasticity (Azcoitia et al., 2017). In contrast, inhibition of aromatase has been shown to interfere with electrophysiological parameters of memory in female mice (Vierk et al., 2012). This is important because the local action in the brain may be poorly related to the circulating levels of the hormone (Giatti et al., 2015). Moreover, the local action of estradiol is different between males and females (Vierk et al., 2012; Melcangi et al., 2016; Ruiz-Palmero et al., 2016).

Observations in humans support the role of estrogens with a special focus on events around menopause. The conception that women have an increased risk of developing cognitive decline and Alzheimer's Dementia (AD) compared to men is widespread in the literature. As suggested above, such increased risk might be due to a reduction in the neuroprotective effects of estrogens on the brain around and following menopause. This view is supported by, for example, findings suggesting that ovariectomy in pre-menopausal women significantly increases risk for the development of memory problems and AD in later life (Ryan et al., 2014). In the same way, other studies have shown that induced hypogonadism, by either surgery or drugs, is accompanied by some detectable decline in cognitive performance (Schmidt et al., 2013).

Together with the above-mentioned observations on the trophic effect of estradiol on synaptic circuitry, classical experiments have shown that this steroid increased the synthesis of choline acetyltransferase (ChAT) in the medial septal nucleus, nucleus basalis of Meynert, and the frontal cortex (Luine, 1985). The potential impact of these findings should be interpreted in terms of the well-established key function of acetylcholine which is a key neurotransmitter in the regulation of surveillance, attention and activation levels, and in memory function, mainly in working and episodic memory (Hasselmo, 2006). The central effects of anticholinergic impacts are generally manifested in behavior with characteristic syndromes such as loss of memory and attention, confused speech and ataxia, confusion, disorientation, and delirium, cognitive impairment or dementia (Campbell et al., 2009).

The alternative hypothesis is that it is not decline in estrogens, but the concomitant increase in GnRH is responsible for neurodegeneration (Skinner et al., 2009). There are GnRH receptors in the human hippocampus, and GnRH is likely to be elevated as a result of the reduction of negative feedback of estrogens after the menopause. To determine whether the elevated level of gonadotropins has an effect on the risk of AD, GnRH analogs (GnRHa) were used in an aged transgenic mouse model. GnRHa produced an acute decline in ovarian hormone output leading to a status similar to menopause. The decrease in LH by GnRHa resulted in an attenuation of amyloid-beta deposition as compared to placebo treated animals. Also, this reduction correlated with improved cognition (Casadesus et al., 2006). Despite the data in support of a role for gonadotropins, most accumulated evidence is concerned with an estrogen-mediated protective effect.

Furthermore, insulin and insulin-like growth factor 1 (IGF1) could play major roles as regulators of growth and regeneration in the CNS. For example, it has been hypothesized that exposure to estradiol may favor the action of IGF1 that, in turn, increases performance of ERα for extended periods, thus favoring protection of hippocampal functions. Specifically, estrogens regulate different insulin-related processes with cognitive correlates (glucose transport, aerobic glycolysis, and mitochondrial function). Consequently, decline in circulating estrogens during menopause is parallel to a decrease in brain bioenergetics and shift toward a metabolically compromised phenotype (Rettberg et al., 2014).

## Clinical studies

Memory complaints are a frequent concern in people older than 50 years, but the cognitive issue in menopausal women is much broader and affects cognitive functioning in general. Memory has been the most studied function, perhaps because it is more clearly affected and because has the potential to predict the development of Alzheimer's disease (AD) (Parikh et al., 2014; Mueller et al., 2015). Studies have addressed changes in subtypes of memory such as verbal recall and working memory, processing speed, attention and executive function, reasoning, difficulty in concentrating, and learning processes.

The impact of the reduction of circulating estrogens on cognitive deterioration has been widely investigated at a clinical level. The effects of natural changes in estrogens and the impacts of interventions such as hormone therapy will be considered separately in the following sections.

## Studies of the relationships between estrogens and cognitive health in peri- and post-menopausal women

A range of methodologies have been used to examine this issue. First, longitudinal or cohort studies have observed and measured both cognition and hormonal status and/or menopausal status over a period of time in the same women—that is, a within participants design, so that the same women are assessed at different time points. Second, cross-sectional studies have been conducted where in-depth data on large groups of women are examined at one point in time with comparisons between women of different menopausal status, or correlations within women between hormonal and cognitive status. Finally, intervention studies, such as randomized controlled trials, have examined the impact of either induced estrogen depletion (for example, in women undergoing clinical procedures) or hormonal therapy (HT) on cognition.

## Cohort studies

The Kinmen Women-Health Investigation (KIWI) (Fuh et al., 2006) is a longitudinal population-based study of rural women in Taiwan. During a follow-up period of 18 months, 114 out of the 495 women progressed to peri-menopause. Women taking HT or who had a history of hysterectomy were excluded from the study. Measures of verbal and visual memory, verbal fluency and the Trail Making test (both generally accepted as indicators of executive functions) and digit span (with forward span indicating immediate short term memory and backward span indicating working memory) were used to assess cognitive performance. Cognitive scores slightly improved for visual recognition, verbal fluency, Trail making A and B, and forward digit span for the women who stayed pre-menopausal. For those who became peri-menopausal, trail making A and B, and forward digit span also improved. However, given the normal influence of practice effects in healthy participants, improvement is not unusual. The important comparisons here are the differences in longitudinal effect between the women who stayed pre-menopausal and those who became peri-menopausal. The researchers controlled for any age differences, education and baseline performance, and found no differences between the two groups of women at follow-up except in verbal fluency, in which there was a significant reduction for the peri-menopausal women. While carefully constructed to control for cohort effects which may be present in cross-sectional studies, and using a limited time gap between testing (20 months) to ensure women were assessed pre-menopause and around the time menopause was beginning, this meant that this study was conducted over a limited time period which may not have been long enough for any negative effects of reduced estrogens on cognition to be clear. In terms of the women's self-perceived health, the percentage of women with excellent or good health decreased after menopause (70.2 vs. 57%), while those who thought their health was poor increased from 2.6 to 4.4%. The authors indicated that the average duration of peri-menopause was nearly 4 years.

The 14 year longitudinal Penn Ovarian Aging Study (POAS) (Epperson et al., 2013) examined women who were pre-menopausal and aged 35–47 at the beginning of the study, with approximately yearly assessments. By the 14^th^ assessment, most women were post-menopausal, with menopausal stages assessed at each visit, using standard menstrually defined menopausal staging categories (Gracia et al., 2005). Differences across menopausal stages were adjusted for age, ethnicity, BMI, education and baseline task performance, and significant reductions in both immediate and delayed recall measures were found. Using menopausal stages as opposed to time as the repeated measures factor enabled these researchers to reveal differences in cognitive effects at different stages of menopausal progression, such that immediate recall declined in the post-menopausal stage but decline in delayed verbal recall occurred in early transition. There were impacts of menopausal stage on measures of processing speed (digit symbol substitution test) and sensorimotor processing speed (symbol copy task) but this was only significant in the unadjusted models, suggesting that age, education, ethnicity, and BMI (as a proxy for health factors such as hypertension, diabetes, and vascular disease) were more important predictors of processing speed than menopausal stage. In the unadjusted model, higher estradiol was associated with better memory performance, but not once data were adjusted for the covariates of age, education, ethnicity, and BMI.

Another longitudinal study, the Seattle Midlife Women's Health Study (SMWHS), explored memory functioning and found that it was more closely related to perceived health, depressed mood or stress than to peri-menopause or age (Woods et al., 2000).

## Cross-sectional observational studies

The Study of Women's Health Across the Nation (SWAN) (Santoro and Sutton-Tyrrell, 2011) is a multi-center, multi-ethnic longitudinal study designed to characterize the physiological and psychosocial changes that occur during the menopausal transition and to observe their effects on subsequent health and risk factors for age-related diseases. In the study, a total of 3,302 women were enrolled at seven clinical sites between 1996 and 1997. At the time of enrollment, women were pre-menopausal, not taking hormones and between 42 and 52 years of age. Participants self-identified as African-American (28%), Caucasian (47%), Chinese (8%), Hispanic (8%), or Japanese (9%). SWAN has a multi-disciplinary focus and thus has repeated measures of bone health, cardiovascular risk factors, psychosocial factors, and ovarian hormones.

Luetters et al. (2007) performed a cross-sectional analysis of a consistent cohort of 1,657 women in the SWAN cohort for whom menopausal status was assessed together with hormonal levels, estradiol and follicle stimulating hormone (FSH). The sample of women was stratified according to menopause stage, and their cognitive function was evaluated. Given that previous studies have been equivocal in terms of whether natural estrogen decline through menopausal stages is related to cognitive decline, this cross-sectional study sought to determine the extent to which menstrually defined menopausal stages were related to cognitive function, but also whether menopausal symptoms, such as hot flushes or poor sleep, mediated any relationship. The study also examined the extent to which cognitive difficulties are related to levels of estradiol or FSH. Researchers assessed immediate and delayed verbal recall, working memory, and processing speed and found no relationships with menopausal stage or hormone levels once sociodemographic factors, self-perceived health, and possible menopausal symptoms were adjusted for. This did not change when either estradiol or FSH levels were included in the analysis. The authors discussed possible limitations of using menstrually defined menopause stages in such analyses, given that there is not a monotonic decline in estradiol levels across stages. For example, there are significant periods in the peri-menopausal stage with heightened estradiol compared with pre- and post-menopausal women (Santoro et al., 1996).

Taken together with the results of other well-controlled studies including those in which many more measures of hormones in the same women were taken across pre- to post-menopause in the longitudinal studies discussed above, findings from the SWAN study suggest that there is no direct effect of the menopause or circulating estrogens on cognition for the measures used. The only clear effect in a well-controlled study was the effect of a decline in verbal fluency in Fuh et al. (2006). Most studies have focused on verbal memory (immediate and delayed) and processing speed, which is appropriate given commonly found age effects on both, but also evidence of specific ERs in the hippocampus. However, few studies have focused on executive function, which is surprising given the evidence for ERs in the prefrontal cortex. Of the tests conducted by the studies reviewed, only verbal fluency and the Trail Making test tap executive function. However, the executive functioning component of trails is calculated by subtracting time taken to complete test A from time taken to complete test B, to subtract psychomotor time from the extra time it takes a participant to alternate between numbers and letters, that is, to inhibit prepotent associates and update one's place in a sequence, the executive components. The Fuh et al. (2006) study did not do this. As such, it seems that further research on the role of menopausal stages and estrogens in effect on executive function, controlling for appropriate covariates, is still needed.

## Interventions: the impact of HT on cognition

Hormone therapy consists predominantly of estrogens, since menopausal symptoms essentially derive from the effect of withdrawal of estrogens at different target tissues. Nevertheless, the normal ovarian cycle includes the regular secretion of progesterone during the luteal phase. Progesterone has a neutralizing effect over endometrial proliferation; for this reason progestogens are added to HT formulations in order to protect the endometrium. So, guidelines recommend that women with a uterus use estrogens plus progestogens, which may be combined in different forms. And because of that, the impact of HT on health should also consider the specific effects of both estrogens and progestogens in comparison to effects of only estrogens.

Observational studies and, more recently, randomized controlled trials have provided current clinical knowledge. The specific form of HT, including hormone preparations, either estrogens alone or combined with progestogens, and the route of the medication, either oral or transdermal have also been investigated. A significant intervention study is the Women's Health Initiative Memory Study (Coker et al., 2010). Conceived as an ancillary study of the Women's Health Initiative randomized controlled trial, the memory study aimed to evaluate the effect of replacement with estrogens plus progestogens on the incidence of dementia or mild cognitive impairment compared with placebo (Shumaker et al., 2003). At the early termination of the study after an average follow up of 4.05 years, 66% of women were diagnosed with probable dementia in the treatment group vs. 34% in the placebo (95% confidence interval; *P* = 0.01). The hazard ratio was 2.05. The incidence of mild cognitive impairment did not differ between groups. This data was corroborated by an MRI study in which regional brain volumes were measured in a subset of 1,403 women, including hippocampal and frontal regions by magnetic resonance at an average of 3.0 years post-trial (Resnick et al., 2009). The use of hormones, both CEE alone or associated with medroxyprogesterone acetate (MPA), was associated with greater brain atrophy, the effects being more evident in women who already demonstrated some cognitive deficits before initiating HT (Resnick et al., 2009). A related study, the Women's Health Initiative Study of Cognitive Aging analyzed the impact of HT on working, verbal and figural memory, speed of processing, attention, executive function, spatial reasoning, and motor performance. They found that the combined estrogens + progesterone formulation had a negative impact on verbal memory but only after long-term HT, with no other cognitive impacts shown. Estrogens alone were associated with lower spatial processing but this effect diminished after a longer duration of therapy (Resnick et al., 2004).

There has been debate on whether such unfavorable impacts might be due to the relatively advanced age, 65 years or older, of participants in the Women's Health Initiative Memory Study. An interpretation of a diminution in the responsiveness of neurons to estrogens with increasing age or the incapacity of the hormone to reverse neural loss and/or dysfunction, which may have occurred during the interim between menopause and the beginning of treatment, cannot be discarded. This effect was examined in the Kronos Early Estrogen Prevention Study (KEEPS), another randomized controlled trial that had an ancillary study, the KEEPS-Cog. Women in this study were younger, 52.6 years average age, and 1.4 years since menopause. A total of 693 women were randomized to daily oral estrogens (CEE) or transdermal estradiol, in both cases associated with micronized progesterone (12 days per month), or placebo. The primary outcome was the effect on the Modified Mini-Mental State examination. Again, HT was not associated with clear benefit, although in contrast to the results in the Women's Health Initiative Memory Study, no harm was found this time (Gleason et al., 2015).

The Women's Health Initiative Memory Study of Younger Women (Coker et al., 2010) had a similar purpose. Cognition was assessed in women who had enrolled in the Women's Health Initiative study when they were 50–55 years of age. When 7.2 years had elapsed since the end of the trial, women were assessed by telephone, their mean age being 67.2 at that moment. As for the KEEPS-Cog, no substantial difference was found between women receiving hormones and the placebo-treated controls (Espeland et al., 2013).

Studies which explicitly examined the impact of years since menopause on the cognitive effects of HT with estradiol are those by Dunkin et al. (2005, 2006). Dunkin et al. (2006) reviewed the literature on the basis of age of the women involved in HT trials and determined that those studies where the women were over the age of 65 have generally failed to find any cognitive benefit, or have found a cognitive disadvantage of HT, whereas those studies using younger participants have been more likely to find positive effects. Indeed, in Dunkin et al.'s randomized placebo-controlled design (Dunkin et al., 2005), these researchers found that recently post-menopausal women benefitted more in terms of executive functioning than women with a longer time since menopause, from a 10 week transdermal estradiol intervention. Some of the latter group actually showed decline in executive function. While these studies showed a significant relationship between years since menopause and cognitive impact of estradiol therapy, with younger women showing a greater positive effect, they did not attempt to disentangle the age and years since menopause confound. Nevertheless, evidence from the Cache County longitudinal study examining HT (Zandi et al., 2002) found that the normally found increased risk of AD in women in comparison to men was not found for women who had begun HT at earlier ages and had used it for around 10 years. The current average age of the women in this study was 73 years, and current users of HT who had not begun it at earlier stages did not show reduced risk of AD.

This accumulation of evidence has led some researchers to propose a critical period for any HT-related neuroprotection (Resnick and Henderson, 2002; Schneider, 2004). Genazzini et al. (2007) suggest a mechanism whereby aging affects ERs, and ER co-activators such as growth factors, neurotransmitters, and neuromodulators. For example, they refer to increased expression of Brain Derived Neurotrophic Factor (BDNF) in young rats in response to estrogens, but decreased BDNF expression in older rats. Given that BDNF has been related to maintenance of brain plasticity in the aging brain in animals and humans, this illustrates the issue. A review by Maki (2013) examines a range of study designs to collate findings on this issue, conducting meta-analyses where appropriate. She concluded that observational studies and neuroimaging studies provide reliable evidence that early use of HT is protective of cognitive function and later use is not, or is detrimental. RCTs showed a benefit or early use of estrogens but not for estrogens + progesterone, regardless of timing and determined that overall there is evidence of both cognitive benefit and reduced risk of AD where the HT is taken in the first 5 years after the last menstrual period when the regimen is not continuous estrogens + progesterone. However, this author also highlighted the possibility that the relationship maybe related to the health of the woman's brain as opposed to their reproductive age, given that data from the Women's Health Initiative Memory Study showed the greatest hippocampal loss in women with initially low cognition scores (Resnick et al., 2009).

Some research has pointed out that, despite the evidence obtained in randomized studies, duration of therapy may have been missed as a variable. Most studies only followed participants for a few years. In response to this uncertainty, the duration of HT was used as a variable in a French cohort assessed for cognitive function. HT was found be associated with better performance in some cognitive domains, such as verbal fluency, visual memory, and psychomotor speed, but only if treatments lasted over 10 years (Ryan et al., 2009). However, if data were adjusted by age, education level, age at menopause, depressive symptoms, etc., none of the effects of HT remained significant. This conclusion is further supported in another recent study in Finland (Imtiaz et al., 2017); in this cohort study, women had received HT from zero (never received it) to more than 5 years and the overall findings provided no strong evidence for a protective effect of estradiol-based HT against cognitive decline.

A further study by Erickson et al. (2007) examined this issue explicitly in relation to cognitive assessments and regional atrophy using detailed voxel based morphometry of MRI scans, enabling analysis of systematic variation of gray and white matter in significant brain regions. They compared HT treatment duration groups of women, balanced for numbers taking opposed or unopposed estrogens, on a test of executive function, the Wisconsin Card Sorting Task, performance on which has been previously shown to be related to prefrontal cortex gray matter. They also used a test of general cognition, the Modified Mini-Mental State Examination. The duration groups were (i) never used HT (ii) up to 10 years (iii) 11–15 years (iv) 16+ years, with a mean age of 69.61 years. Given that both physical exercise and estrogens have similar actions on neurotrophic growth and vascularization, and BDNF actions are affected both by estrogens and exercise in the hippocampus, with humans showing the greatest concentration of BDNF receptors in the frontal lobes, Erickson et al. (2007) hypothesized that estrogens and exercise may interact to positively affect human brain tissue in the frontal lobes and also cognition. They found that HT duration up to 10 years was associated with better performance on the Wisconsin Card Sorting Task and with spared gray matter in the prefrontal cortex, compared with other groups, adjusting for age, age at menopause, socio-economic status and education. However, HT duration longer than 10 years was associated with greater prefrontal deterioration and greater decline in the measures of executive function. Importantly, physical fitness level (measured by VO_2_ max) interacted with duration such that higher fitness levels ameliorated the negative effects of longer duration of HT and enhanced positive effects of shorter durations in both performance and brain morphometry. This evidence for interactions between estrogen therapy and exercise/fitness on cognitive and brain health is important, and supports the role of healthy lifestyle interventions alongside HT. However, the effects were specific to executive function and did not occur for the general cognition measure.

To summarize, and in consistence with the conclusion from other groups (Henderson and Popat, 2011), the most solid evidence seems to favor the notion that HT is not indicated for treatment of cognitive decline or dementia, and that the inconsistency in results may be related to the control of confounding variables, the timeline (onset and duration) of the use of HT and to the treatment regimen. Maki (2013) suggests that there is tentative support for beneficial effects for hysterectomised women who are treated with estrogens alone, and Erickson et al.'s (2007) suggests durations longer than 10 years are not beneficial but that HT benefit or negative effects interact with physical fitness and health. The concept of a critical period of treatment in early post-menopause seems well-established as at least not a disadvantage for cognition. However, studies are still insufficient to be clear on which circumstances, to which women, and for how long HT should be prescribed for maximum cognitive benefit and/or minimum cognitive negative effect. Studies where the underlying brain health or physical fitness of the women is considered as a variable, seem a useful way to move forward.

## Mood and cognition

The influence of mood, particularly depressed mood and anxiety, on cognition is supported by considerable evidence at all ages, but a greater impact of depression on cognition is recognized for older people. Indeed, higher levels of depressive and anxiety symptoms are directly related to poorer cognitive performance because both disorders are often accompanied by attention and concentration deficit symptoms. There is considerable support for depression as a risk factor for cognitive impairment, with Chung et al. (2015) finding an association between lifetime history of major depression and amyloid beta deposition in individuals with mild cognitive impairment (MCI). In addition to the influence of depression on cognition, depression is commonly implicated as a significant risk factor for dementia in older adults (da Silva et al., 2013) to the extent that it may be a prodromal symptom of some kinds of dementia (Panza et al., 2010). That is, depression can be both a risk factor for dementia and an early indicator of incipient dementia (Fiske et al., 2009). Both anxiety and depression affect cognitive performance at all ages, with long term anxiety being a risk factor for depression. However, stress also has a long-term negative effect on cognition, particularly executive function. Stress affects similar brain structures and cognitive functions (particularly memory) as advancing age, e.g., the hippocampus or the frontal lobes, with effects of psychological stress mediated by the effects of the action of stress hormones such as glucocorticoids (Bunce et al., 2008).

The correlation of depressed mood with nearly every indicator of memory functioning, for example, in the longitudinal studies discussed above (Woods et al., 2000), raises important questions and opens the door to indirect actions of estrogens on cognition through their well-known relationship with mood states. The influence of ovarian function on mood states has been widely claimed in the literature for a considerable time. The issue is interesting in itself, but also in terms of its effects on cognition in later life. That is, mood state could act as an indirect effect of changes in sex hormones on cognition with increasing age. Thus, cognitive complaints during peri-menopause may at least partially result from the effect of peri-menopausal anxiety or depressive symptoms on the brain (Greendale et al., 2011).

Research has demonstrated that the lifetime prevalence rate of mood disorders is significantly greater in women than in men, approximately two times more frequent (Walf and Frye, 2006; Ter-Horst et al., 2009). Data suggests that estrogens, or their absence, are strongly implicated in the regulation of mood and behavior, as well as in the pathobiology of mood disorders (Halbreich and Kahn, 2001). So the interaction of estrogens with the serotonergic systems (Amin et al., 2006) has been taken to propose estradiol as a protective agent against mood changes related to serotonin withdrawal (Wharton et al., 2012). Due to the correlation between depression and serotonin, estrogen treatment can have beneficial effects to buffer changes in mood.

Clinical observation also shows that estrogens play an essential role in the emergence and course of mood disorders in women. Gender differences in mood disorders are present throughout the lifespan, but periods of hormonal fluctuations or estrogens instability (i.e., pre-menstrually, post-partum, peri-menopausally) are related to increased vulnerability to mood disorders among susceptible women (Halbreich and Kahn, 2001).

Mood disorders are prevalent during the menopausal transition, with prevalence figures of 16.5% for depressed mood in women during midlife (Prairie et al., 2015). The SWAN study found that women were two to four times more likely to suffer a depressive episode during the menopausal transition than during the pre-menopausal period (Bromberger et al., 2011). Other investigators have reported similar findings (Soares, 2010). The known interaction of estrogens with serotonin and other monoamines provides biochemical rationality to the clinical findings (Warnock et al., 2017). Mood problems have been found to increase in those with a history of mood disorders, but can also occur *de novo* as a consequence of the hormonal changes. In most cases, the period of vulnerability to mood problems (Alexander et al., 2007) subsides when hormonal levels stabilize and women enter full menopause. It is understood that it is hormonal fluctuations that more directly determine the mood changes (Wharton et al., 2012).

## HT and mood

The clear hormonal influence in mood fluctuations has prompted the postulation of HT as a useful remedy. One recent central study is the previously mentioned KEEPS-Cog, a multicenter randomized trial that, in addition to cognitive effects, assessed the impact on mood, including depression and anxiety. Oral estrogens (CEE), but not transdermal estradiol, effectively reduced scores of depression and anxiety over the 48 months of treatment (Gleason et al., 2015). The reasons for the difference between the treatments remain elusive, although it is possible that the maintained levels of circulating estradiol downregulates ER in the concerned brain regions. This effect of estrogens in KEEPS-Cog further confirms previous studies of different sizes and relevance, as shown in several reviews (Schmidt et al., 2000; Soares et al., 2001; Schiff et al., 2005). Fischer et al. (2014) reviewed five RCTs which investigated the impact of HT on mood, all of which showed a benefit of treatment. Three of the studies involved only estradiol, whereas two found effects for both estrogens alone or with different amounts of progesterone (MPA) and a further study found reductions in depression and anxiety following 3 months of transdermal estradiol with or without norethisterone. Thus, although there is a suggestion of positive effects on mood of HT, the mechanisms need further investigation, with remaining questions being the extent to which mood improvements are direct effects, or indirect via effects of HT on other distressing menopausal symptoms such as sleep disturbance or hot flushes. Furthermore, the complex relationship of depression with cognition also suggests that the influence of HT on depression as a possible mediator of any observed influence on cognition could also be examined in future research, rather than as separate effects.

## Indirect impacts on cognition via effects of sex hormone reduction on cardiovascular health

Cardiovascular diseases represent an important health issue all over the world because they are the primary cause of death for both men and women. Morbidity and mortality rates are higher in men before menopause, whereas after this age, these rates converge.

As reviewed in Navarro-Pardo et al. (2017), estrogens act to improve lipid profile, promote vasodilatation and antioxidant activities, supporting vascular health. By contrast, the menopause leads to an overturn of all these effects, increasing cardiovascular risk. Vascular deterioration is an important issue that should be considered when assessing the impact of the menopause on cognition, for example, the vascular changes associated with vasomotor symptoms that take place during and after menopause. The SWAN study evaluated whether the decrease in cognitive processing speed observed during peri-menopause might be influenced by the existence of hot flushes. Moreover, anxiety and depressive symptoms were also considered in the research. Only a small effect was found, related to anxiety and depressive symptoms, but there was no interaction with vasomotor symptoms (Luetters et al., 2007).

A possible long-term effect has also been raised. The hypothesis that the menopause might accelerate atherosclerosis and consequently increase future cardiovascular risk, has a correlate in terms of cognitive function. Vascular factors are involved in roughly half of all dementia cases, probably because they show the cumulative impact of very different factors along lifespan (Bowler, 2005; Sonnen et al., 2007; Gorelick et al., 2011). Outside of dementia, vascular factors are also associated with cognitive change in otherwise healthy older people. Going back to early longitudinal studies, Wilkie and Eisdorfer (1971) pointed out that “The presence of large numbers of aged with cardiovascular illness suggests that the basis for the cognitive decline associated with aging after maturity should be considered secondary to some pathologic process, and not merely as a “normal” aging process” (p962) and longitudinal studies such as Okonkwo et al. (2011) demonstrated that cardiovascular indices such as variability in blood pressure and cardiac output predicted declines in attention, executive function and psychomotor function over 36 months, but basic resting blood pressure measurements did not. Likewise, Singh-Manoux et al. (2003) demonstrated prediction of poor cognitive function from occurrence of angina pectoris, myocardial infarction, all coronary heart disease and intermittent claudication, 11 years prior to the cognitive measures.

The notion of a critical period for positive cognitive effect or avoidance of a negative impact on cognition or increase in dementia risk is mirrored in investigations of HT where HT is shown to be beneficial in the early peri- and post-menopausal period, but not in older women or those who may already have advanced atherosclerosis (Hodis et al., 2016; Savolainen-Peltonen et al., 2016). These parallel findings further suggest the role of estrogens effects on the cardiovascular system as a mediator in the impact on cognitive function health and changes.

Thus, the relationship between cardiovascular health and cognitive health is well-supported, but studies examining the impact of HT on cardiovascular health as a mediator of any beneficial impact on cognition are needed.

## Conclusions

The presence of ERs has been confirmed in different brain locations related to cognition, mainly to verbal memory and retrieval, working memory, executive function and attention, such as the hippocampus and the prefrontal cortex, leading to the rationale that changes in levels of estrogens would produce direct effects in the brain and accordingly in cognitive function. Furthermore, the interaction of estrogens with serotonergic pathways also provides a focus for explaining peri-menopausal mood changes.

Menopausal withdrawal of estrogens could also have a specific impact on the vascular tree and thereby have a secondary effect on cognition such as memory declines via vasomotor symptoms.

A range of methodologies have been used to study these effects, such as longitudinal, cross-sectional, and intervention studies, including randomized controlled trials. In reviewing several of these studies, it is concluded that menopause effects on cognitive functioning are not clear because of the series of confounding variables found to have an effect. When confounds such as age are controlled, the most reliable effects are for a measure related to executive function, verbal fluency.

Research on hormonal therapy (HT) in menopausal women has also been reviewed. Differences in HT regarding therapy timeline (onset and duration), composition, manner of administration, and interaction effects with other variables (such as age differences, education, and baseline performance) had significant impacts and made direct comparisons amongst the studies difficult.

It was concluded that HT does not have a clear impact on cognition, including episodic memory or executive functions and is not recommended as a treatment or prevention pathway for cognitive decline or dementia in older women. There is not enough evidence to support the existence of a window of opportunity in the immediate post-menopause, but an HT neutral effect (on cognition) extends for a reasonable number of years post-menopause. The suggestions of beneficial effects on cognitive function in the first few years after menopause may imply some risk reduction regarding cognitive decline and dementias. Although there are useful developments, for example, the interaction of HT impacts with the benefits of physical exercise, late-life cognitive consequences are still poorly addressed.

On another front, mood disorders are twice as frequent in women than in men, and estrogens changes are probably implicated given that differences appear from menarche through reproductive age, including periods of estrogenic instability. An important component of well-being, self-perceived health, seems to change significantly from pre- to post-menopause. Estrogens interact with the serotonergic system so that estradiol could have a protective effect against mood changes, which could implicate the reduction in sex hormones post-menopausally in related episodes of low mood and depression. Related to this depression-serotonin correlation, HT could have a beneficial effect on mood disorders, although the mechanisms need further investigation. Depressed mood and anxiety have a negative influence on cognition, particularly on memory functioning, particularly so for older people. Depression is a well-known risk factor or prodromal symptom for cognitive impairment and dementia.

Cardiovascular effects of reducing estrogen, including increased cardiovascular risk, seem to act as a secondary pathway by which reductions in this sex hormone has an impact on cognition, for example, given that vascular effects are implicated in at least half of all dementia cases and in many other aging processes.

## Author contributions

EN-P, CH, and AC: have contributed equally to this article. All authors have read and approved the final manuscript.

### Conflict of interest statement

The authors declare that the research was conducted in the absence of any commercial or financial relationships that could be construed as a potential conflict of interest.
